# Current evidence on basic emergency obstetric and newborn care services in Addis Ababa, Ethiopia; a cross sectional study

**DOI:** 10.1186/1471-2393-14-354

**Published:** 2014-10-10

**Authors:** Alemnesh H Mirkuzie, Mitike Molla Sisay, Alemnesh Tekelebirhan Reta, Mulu Muleta Bedane

**Affiliations:** Centre for International Health, University of Bergen, Årstadv 21, Overlegedanielsenshus, Bergen, 5020 Norway; School of Public Health, College of Health Sciences, TikurAnbessa Hospital, Second Floor, Room Number 58, PO Box 40860, Addis Ababa, Ethiopia; Jhpiego Ethiopia, MNCH, Addis Ababa, Ethiopia; WAHA International, University of Gondar, Post box 41822, Gondar, Ethiopia

**Keywords:** Assessment, Basic, Care, Emergency, Knowledge, Neonatal, Obstetric, Skills

## Abstract

**Background:**

Emergency obstetric and neonatal care (EmONC) is a high impact priority intervention highly recommended for improving maternal and neonatal health outcomes. In 2008, Ethiopia conducted a national EmONC survey that revealed implementation gaps, mainly due to resource constraints and poor competence among providers. As part of an ongoing project, this paper examined progress in the implementation of the basic EmONC (BEmONC) in Addis Ababa and compared with the 2008 survey.

**Methods:**

A facility based intervention project was conducted in 10 randomly selected public health centers (HCs) in Addis Ababa and baseline data collected on BEmONC status from January to March 2013. Retrospective routine record reviews and facility observations were done in 29 HCs in 2008 and in10 HCs in 2013. Twenty-five providers in 2008 and 24 in 2013 participated in BEmONC knowledge and skills assessment. All the data were collected using standard tools. Descriptive statistics and t-tests were used.

**Results:**

In 2013, all the surveyed HCs had continuous water supply, reliable access to telephone, logbooks & phartograph. Fifty precent of the HCs in 2013 and 34% in 2008 had access to 24 hours ambulance services. The ratio of midwives to 100 expected births were 0.26 in 2008 and 10.3 in 2013. In 2008, 67% of the HCs had a formal fee waiver system while all the surveyed HCs had it in 2013. HCs reporting a consistent supply of uterotonic drugs were 85% in 2008 and 100% in 2013. The majority of the providers who participated in both surveys reported to have insufficient knowledge in diagnosing postpartum haemorrhage (PPH) and birth asphyxia as well as poor skills in neonatal resuscitation. Comparing with the 2008 survey, no significant improvements were observed in providers’ knowledge and competence in 2013 on PPH management and essential newborn care (p > 0.05).

**Conclusion:**

There are advances in infrastructure, medical supplies and personnel for EmONC provision, yet poor providers’ competences have persisted contributing to the quality gaps on BEmONC in Addis Ababa. Considering short-term in-service trainings using novel approaches for ensuring desired competences for large number of providers in short time period is imperative.

## Background

Emergency Obstetric and Neonatal Care (EmONC) is a cost effective priority intervention to reduce maternal and neonatal morbidity and mortality in poor resource settings [1, 2]. Basic EmONC (BEmONC) alone can avert 40% of intrapartum related neonatal deaths and a significant proportion of maternal mortality [3]. Although EmONC initiatives have been implemented in Ethiopia since 1998 [4], the services were not widely available and were inaccessible to the women who needed the services according to a national EmONC survey conducted in 2008 [5]. All the regions are characterized by poor availability and accessibility of the EmONC services, but Addis Ababa and Harere. Weak infrastructure and resource limitations coupled with terrine geography and sparse distributions of rural communities are implicated for the poor availability and accessibility of EmONC in the regions.

In contrast, Addis Ababa has fulfilled the WHO minimum standard in terms of availability and accessibility of the EmONC services yet the quality of the services remains highly compromised. In 2008 there were 29 Health Centres (HCs) providing BEmONC services in Addis Ababa. However, none of these HCs were fully functional, as they had not performed one or more signal function in the three months preceding the survey [5]. Complying with recommendations from the 2008 national EmONC assessment, efforts have been made by local and international stakeholders to improve the quality of EmONC services and to reduce the burden of maternal and neonatal morbidity and mortality.

A hospital based national survey in 2010 that analysed constraints from policy to practice in selected emergency obstetric and neonatal conditions in 18 hospitals identified providers poor competence as a major deterrent for ensuring quality comprehensive EmONC [6]. In the case of postpartum haemorrhage (PPH) for instance, all the hospitals had policy for PPH management, 92% had the required health personnel, 96% had the necessary supplies and 64% received supervision; yet only 39% of the providers in these hospitals had good knowledge on PPH and only 29% had the skills to properly manage PPH. The same was true for essential newborn care whereby all the hospitals had the policy, 92% had the required health personnel, 70% had the necessary supplies and 64% received supervision; however only 55% of the providers had sufficient knowledge with only 18% providing the care.

Acknowledging the persistence of poor competency among obstetric and newborn health care providers, in 2010 the Federal Ministry of Health (FMOH) in partnership with local and international stakeholders developed standard BEmONC curricula for in service training [7]. The curricula is a comprehensive document covering a wide range of topics complemented with up-to-date training approaches and gives due emphasis on high impact priority interventions for maximum health gains. Hence, there is a need to revisit the implementation of the BEmONC initiative in Addis Ababa and the progresses made. Making comparisons between the current EmONC status and the 2008 are also important to identify and to respond to the remaining challenges. Findings informed a collaborative project between the University of Bergen, the Addis Ababa City Council Health Bureau and the Addis Ababa University to improve the quality of BEmONC in Addis Ababa. The project uses a before-after design to document changes in maternal and neonatal outcomes. In this paper, we present the baseline data on the BEmONC status of the 10 project HCs in 2013. The paper gives special focus on PPH and neonatal care as these two conditions are well described in the 2008 EmONC survey, which would facilitate comparison between the two surveys and help to document progresses made since 2008.

## Methods

### Study settings

Currently, over 70 public HCs and 4 public hospitals under the Addis Ababa City Administration, Health Bureau provide maternal and child health services to about 80% of the population while the private health facilities share is only about 20% of the care. BEmONC services are provided in the public HCs and hospitals provide comprehensive EmONC. Seven signal EmONC functions are provided at the BEmONC facilities which include parenteral antibiotic, parenteral uterotonic, parenteral anti-convalescent, assisted vaginal delivery, manual removal of placenta, removal of retained product and newborn resuscitation [8]. In addition to the seven signal functions, blood transfusion and caesarean section are provided in comprehensive EmONC facilities. There is a referral network system between HCs and hospitals for mothers and newborn babies requiring advanced interventions. Providers who are referring the mothers or newborn babies arrange ambulance services. The median distance from referring HC to the nearest hospital with surgical service was five about km in 2008 and is expected to be less as the number of HCs has doubled by 2013. All the 10 HCs surveyed in our project in 2013 were also surveyed during the 2008 national EmONC assessment.

### Study design, sampling and data collection

A health facility based intervention project has been implemented to improve maternal and neonatal health outcomes in Addis Ababa. The interventions include 1) intensive hands on skills training using simulation technology, 2) developing diagnostic and management protocol for pre-mature rupture of membranes (PROM) occurring at term and 3) implementing the PROM protocol. Ten public HCs were randomly selected, one from each sub-city. These were Woreda 7 HC from Addis Ketema sub-city, Saris HC from AkakiKality, Kebena HC from Arada, Bole 17 HC from Bole, Shiromeda HC from Gulele, Tekelehymanot HC from Lideta, Meshualekiya HC from Kirkos, Woreda 9 HC from KolfeKeraniyo, Woreda 9 HC from Nifas Silk Lafto and Entoto 1 HC from Yeka sub-city.

Data collection methods include retrospective review of routine records, interviews with providers and facility observations. The principal investigator collected all the data between January and March 2013. Trained professionals did the data collection in 2008 from 29 HCs in Addis Ababa. Standard data collection tools, which were adapted to the Ethiopian context during the 2008 national EmONC survey was used in our survey in 2013 [5]. Four major areas were assessed: 1) identification of facility and infrastructure, using observation and interviewing a person of some authority at the facility 2) human recourses, using interview with one knowledgeable person about the staffing pattern and staffing situation 24 hours/7 days a week in the facility 3) essential drugs, equipment and supplies for the provision of EmONC using observation and interviewing a person of some authority at the facility 4) providers knowledge and competency for maternal and newborn care; 24 providers in 2013 and 25 in 2008 were interviewed to assess their knowledge in diagnosing and managing normal labour, PPH and neonatal conditions. Providers were also asked if they ever received EmONC training. In both surveys, the interviewed providers were selected on the basis of their presence on the date the HCs were visited with random selection in 2013 and those who attended the largest number of deliveries in the 2008 survey.

Five questions on obstetrics and five on neonatal care were asked. For assessing knowledge, under each question a list of correct choices were given and providers were asked to give multiple answers (Table 1). Observation of actual performance when care is provided is the standard method for assessing skills. However, this method was not used in the 2008 survey. To facilitate fair comparison between the two surveys we used the same methodologies that were used in 2008. Hence, the proxy skill assessment method used in both surveys was asking providers what they would do to manage an asphyxiated baby for instance. Another method used to assess skill was asking providers what immediate newborn care they provided the last time they attended birth. In both cases interviewees were asked open-ended questions and were not prompted on specific practices.

**Table 1:** **Questions used to evaluate obstetric and neonatal care knowledge and skills of providers**

Obstetric knowledge and skills	Neonatal care knowledge and skills
**1. Diagnosing labour (4)**	**1. Diagnosing Birth Asphyxia (4)**
*Dilation of the cervix*	*Depressed breathing*
*Regular uterine contractions*	*Floppiness*
*Discharge of blood and mucus*	*Heart rate < 100 beats per minute*
*Breaking of the waters/ruptured membranes*	*Central cyanosis*
**2. Monitoring a woman is in labour (9)**	**2. Preliminary steps of neonatal resuscitation (6)**
*Foetal heart beat*	*Place new born face up*
*Dialation of the cervix*	*Wrap baby, except for face and upper chest*
*Maternal blood pressure*	*Position baby’s head so neck is extended*
*Uterine contractions*	*Aspirate mouth and then nose*
*Maternal pulse*	*Stimulate by rubbing back*
*Maternal temperature*	*Explain process to mother*
*Descent of the head*	**3. Steps of bag and mask resuscitation**
*Colour of amniotic fluid*	*Cover baby’s chin, mouth and nose with mask*
*Degree of moulding*	*Ensure seal*
**3. Steps of AMTSL**	*Ventilate 40 times per min*
*Immediate oxytocin (1 to 2 min)*	*Pause to determine breathing*
*Controlled cord traction*	**4. Care for baby who failed to breathe (3)**
*Uterine massage*	*Continue ventilation with bag and mask*
**4. Observation for PPH (6)**	*Assess need for special care*
*Sign of shock*	*Explain to mother what is happening*
*Signs of anaemia*	**5. Immediate new born care provided (10)**
*Retained products or retained placenta*	*Clean the mouth, face and nose*
*Amount of external blood*	*Ensure the baby is breathing*
*Damage to the genital tract*	*Ensure the baby is dry*
*Whether uterus is contracted*	*Observe for colour*
**5. Care for a woman with PPH (6)**	*Care for the umbilical cord*
*Begin intravenous fluids*	*Provide prophylaxis for eyes*
*Give ergometrine or oxytocin (IV or IM)*	*Weigh the baby*
*Manually remove retained products*	*Thermal protection (skin to skin)*
*Examine woman for lacerations*	*Begin breastfeeding within first hour*
*Massage the fundus*	*Evaluate/examine baby within first hour*
*Refer*	

Percentage, mean scores and fisher exact chi square tests were used for data analyses. We also used independent samples t-tests for comparing knowledge and skills mean scores between the 2008 and 2013 surveys.

The project has received ethical approval from the Addis Ababa City Administration Health Bureau, Ethiopia and the Regional Ethics Committee in Western Norway. Study permits were sought from the Addis Ababa City Administration Health Bureau, the Health Bureaus’ of the respective sub-cities and from all the project health centers. Written informed consent were obtained from the study participants.

## Results

### Infrastructure

Hundred percent of the surveyed HCs had continuous water and electric supplies and delivery logbooks in 2013 and in 2008. By 2013, 100% of the HCs were having reliable access to telephone and partograph while these figures were 24% and 44% respectively in 2008. Ambulance services were available in 50% of the HCs in 2013 while the remaining HCs were relaying on ambulances from the fire department (command post) especially off working hours. In 2008, only 34% HCs reported to have access to ambulance services (Table 2).

**Table 2:** **Distribution of essential facilities in 2013 in the 10 HCs in Addis Ababa**

Health centre	Partograph	Water supply	Generator	Emergency lamp	Telephone	Ambulance	Delivery logbook
**Woreda 7**	✓	✓	✓	X	✓	✓	✓
**Bole 17**	✓	✓	x	✓	✓	✓	✓
**Woreda 9, NSLSC**	✓	✓	x	✓	✓	x	✓
**Saris**	✓	✓	✓	✓	✓	x	✓
**Teklehaymanot**	✓	✓	x	X	✓	x	✓
**Kebena**	✓	✓	✓	X	✓	✓	✓
**Meshualekiya**	✓	✓	x	✓	✓	x	✓
**Shiromeda**	✓	✓	x	x	✓	✓	✓
**Entoto 1**	✓	✓	x	x	✓	✓	✓
**Woreda 9, KKSC**	✓	✓	✓	✓	✓	x	✓
**Total parcentage**	100%	100%	40%	50%	100%	50%	100%

NB: NSLSC stands for Nifas Silk Lafto Sub City, KKSC stands for Kolfe Keraniyo Sub City.

In 2013, all the HCs had a formal fee waiver system and were providing maternal and newborn care free of charge while in 2008, 67% had a formal system to waiver maternal and newborn care.

### Essential medicines and supplies

In 2013, all the HCs had reliable supply (no reporting of recent stock outs) of uterotonic drugs, antibiotic eye ointment and intravenous fluids while 50% had parenteral antibiotics and parenteral anticonvulsant (Valium). In the 2008 survey, 85% of the HCs had a reliable supply of uterotonic drugs and 65% had Valium. None of the HCs had Magnesium Sulfate (MgSO4) both in 2013 and 2008. Ninety percent and 76% of the HCs had a functional vacuum extractor in 2013 (Table 3) and 2008 respectively (data not shown).

**Table 3:** **Shows essential EmONC medicine and supplies available in 2013 in the10 HCs in Addis Ababa**

Health center	Intravenous ampicillin	Eye ointment	Valium	Misoprostol	Ergometrine	Oxytocin	IV fluids
**Woreda 7**	✓	✓	x	✓	✓	✓	✓
**Bole 17**	✓	✓	✓	✓	✓	✓	✓
**Woreda 9, NSLSC**	X	✓	x	✓	✓	✓	✓
**Saris**	✓	✓	x	✓	✓	✓	✓
**Tekelehaymanot**	X	✓	✓	✓	✓	✓	✓
**Kebena**	X	✓	✓	✓	✓	✓	✓
**Meshualekiya**	X	✓	x	✓	✓	✓	✓
**Shiromeda**	✓	✓	✓	✓	✓	✓	✓
**Entoto 1**	X	✓	x	✓	✓	✓	✓
**Woreda 9, KKSC**	✓	✓	✓	✓	✓	✓	✓
**Total percentage**	50%	100%	50%	100%	100%	100%	100%

NB: NSLSC stands for Nifas Silk Lafto Sub City, KKSC stands for Kolfe Keraniyo Sub City.

### Manpower and caseload

In 2013, there were 72 full time providers working in the labour ward in the 10 HCs with a staffing level of minimum 5 and a maximum 12. The midwives outnumber the nurses (52 vs 20). There were a total of 72 obstetric beds and 22 delivery couches and the total number of deliveries attended in the 10 HCs in the month preceding the survey was 505, which ranged from 18 to 122. In 2013, the ratio of providers for 100 deliveries was 10.3 for midwives and 14.2 for a skilled birth attendant (midwives plus nurses) (Table 4). In 2008, this ratio was 0.26 for midwives and 2.88 for skilled birth attendant (data not shown).

**Table 4:** **Distribution of manpower, caseload and obstetric beds in 2013 in the 10 HCs in Addis Ababa**

Health centre	# fulltime professionals in labour ward	Midwives	Obstetric bed	Delivery couch	# deliveries in the last month
**Woreda 7**	6	6	4	2	40
**Bole 17**	10	8	11	2	68
**Woreda 9,NSLSC**	5	3	10	1	52
**Saris**	8	3	8	4	53
**Teklehaymanot**	6	6	4	2	25
**Kebena**	6	6	5	2	18
**Meshualekiya**	5	5	6	2	27
**Shiromeda**	5	5	8	2	33
**Entoto 1**	9	4	8	3	67
**Woreda 9, KKSC**	12	6	7	2	122
**Total**	72	52	71	22	505

NB: NSLSC stands for Nifas Silk Lafto Sub City, KKSC stands for Kolfe Keraniyo Sub City.

### Providers’ knowledge and competence

Of the 24 interviewed providers who were working in the labour ward of the 10 HCs, 21 were midwives and three were nurses. They were between 22 and 36 year of age (mean age 28 years) having five months to 19 years of work experience. Three of the 24 interviewed providers in 2013 and four of the 25 interviewed providers in 2008 reported that they had ever received in-service EmOC training. All the interviewed providers acknowledged that PPH management and neonatal resuscitation are covered in their pre-service curricula. All the 24 providers in 2013 reported that they were routinely using partographs for monitoring and recording labour progress and 44% providers in 2008 reported using partograph.

Table 5 shows providers mean knowledge scores in selected obstetric conditions. In general, providers reported insufficient knowledge both in 2013 and in 2008. Providers who reported to have ever received EmONC training did not demonstrating better knowledge compared to those who never received the training. In 2013, four participants reported that they did not know about Active Management of Third Stage Labour (AMTSL) at all. When we compared the mean knowledge scores of providers on selected obstetric procedures between the 2013 and 2008 surveys, no significant differences were observed (Table 5).

**Table 5:** **Providers mean knowledge scores on diagnosis and management of labour, bleeding after childbirth, birth asphyxia and skill scores on neonatal resuscitation in 2008 and 2013**

Question	2008 n=25	2013 n=24	P-values
How do you know when a pregnant woman is in labour?	3.2	3.1	0.38
**Average score (out of 4)**			
What do you monitor when a woman is in labour?	5.6	6.3	0.12
**Average score (out of 9)**			
What are the steps of AMTSL?	2.5	2.3	0.49
**Average score (out of 3)**			
What do you look for when a woman arrives with or develops heavy bleeding after birth?	3.7	3.8	0.90
**Average score (out of 6)**			
What do you do when a woman arrives with or develops heavy bleeding after birth?	4.6	4.4	0.51
**Average score (out of 6)**			
How do you diagnosis birth asphyxia?	2.2	2.4	0.49
**Average score (out of 4)**			
If resuscitate a neonate with bag mask, what do you do?	2.3	1.5	0.10
**Average score (out of 5)**			

In 2013, providers mean score on diagnosing birth asphyxia was 2.4 out of 4 correct answers. Five providers only knew four of the six preliminary steps of neonatal resuscitation. The mean skill score on how to resuscitate a neonate with bag mask was 1.5 out of 5 correct answers. Comparison of the mean competence score on essential newborn care between the 2013 and 2008 surveys showed no significant difference (p >0.05) (Table 5).

## Discussion

The study has shown that the majority of the health centres in 2013 had the necessary inputs and personnel for the provision of BEmONC which presents a major improvement in the part of the HCs compared with the 2008 EmONC survey [5]. These reflect the efforts made by the government and other stakeholders to bridge quality gaps on BEmONC in Addis Ababa. However, the lack of progresses between 2008 and 2013 in provider’s competence in detecting, preventing and managing emergency obstetric and neonatal complications entails further concerted efforts.

PPH remains a major cause of maternal mortality and morbidity in poor resource settings [8, 9]. In the absence of proper and prompt management, PPH could claim the life of the women within two hours [8]. Our study showed poor provider’s competence on preventing and managing PPH with no progress over the past years. This is consistent with a study in 18 hospitals in Ethiopia, where only 39% of the providers have knowledge to prevent PPH by 2010 [6]. The persistence of poor providers’ competence could be attributed to gaps in pre-service curricula, lack of continuing education, staff turnover, frequent rotation and limited in-service training in Addis Ababa. A multi country study that included Ethiopia and a study from Nigeria report that the pre-service curricula for midwives and nurses have limitations to ensure graduates with essential midwifery competences up to the standard set by the International Confederation of Midwives [10, 11].

Only 12% of the interviewed providers in 2013 and 16% in 2008 reported receiving in-service BEmONC training. The national Health Sector Development Programme for the year 2010 to 2015 has given focus on developing critical work force skills for improving the quality of health care in Ethiopia with due emphasis on standard in-service trainings [12]. In view of that, a UNICEF funded project run by Jhpiego in collaboration with the Federal Ministry of Health (FMOH) provided standard BEmONC training for 2007 providers across the country expect Addis Ababa. This would suggest that there is little strategic focus in Addis Ababa to scale up in-service BEmONC training, which requires prompt attention [13].

Atonic PPH is the commonest cause of PPH and is showing an increasing trend [8, 14, 15]. Active Management of Third Stage Labour (AMTSL) is an effective intervention for preventing and managing atonic PPH [8, 16, 17]. AMTSL has three steps; the first step is the administration of oxytocin within 1–2 minutes of birth; the second step is controlled contraction and the third step is uterine massage. Oxytocin is an essential drug for AMTSL whereby all the HCs in 2013 and 85% in 2008 reported having a reliable supply. However, providers’ knowledge on AMTSL remained sub-optimal both in the 2008 and 2013 surveys. Competence gaps in AMSTL appear to be commonplace in many resource poor settings, despite the availability of essential supplies of the procedure. Studies from eight countries, including Ethiopia have reported that AMTSL use is ranging from 0.5% to 32% [6, 18].

Neonatal asphyxia occurs when there is failure to initiate spontaneous breathing whereby about 10% of newborn babies could have this problem at birth [19]. Hence, competence on neonatal resuscitation is critical to help babies who failed to initiate spontaneous breathing at birth or those who breathe poorly [19, 20]. Our study revealed that providers have limited knowledge and skill in identifying and managing neonatal asphyxia and no improvements have seen in 2013 compared to 2008. Consistent with our findings, in a nationwide survey only 55% of professional providing intrapartum care in hospitals have had sufficient knowledge on essential newborn care with only 18% having ever resuscitated a newborn infant [6]. Babies born asphyxiated should be ventilated within the first minute after birth also called “The Golden Minute” and delays to initiate ventilation increase the risk of mortality and long term neurological sequel [19–21].

In Addis Ababa although about 85% of the births are said to be taking place in the health facilities, by 2012 there were 30 stillbirths for 1000 births [22, 23]. Several studies have documented high correlation between facility births and better maternal and perinatal outcomes, yet the situation in Addis Ababa appears to defy this notion [24]. In a study by Harvey and colleagues; many maternal and newborn care providers are not skilled enough; hence giving birth at a health care facility does not guarantee skilled birth care, which seem to be the case in Addis Ababa [25, 26]. Moreover, some of the intrapartum stillbirths could be attributed to diagnostic bias where babies born with primary apnoea would have been misclassified as stillbirths [20]. Although, Ethiopia has achieved MDG 4, two years ahead of time the neonatal mortality and the rate of stillbirth remains high suggesting the need for quality improvement in maternal and newborn care services with, a focus on in-service EmONC training [27].

Several studies have shown that in-service training on essential newborn care and neonatal resuscitation significantly reduces perinatal mortality [3, 21, 28]. In 2010, the FMOH in partnership with other stakeholders developed standard national BEmONC training curricula [7]. The standard BEmONC curricula cover a wide range of obstetric and newborn care topics to ensure providers with the necessary competences. The training is conducted in special set up for three weeks and currently being scaled up across the country. The training curricula incorporated up-to-date interventions, including Helping Mothers Survive (HMS) and Helping Babies Breathe (HBB) modules that use low cost and low-tech simulators proved effective and efficient for ensuring providers competences [21, 28]. The HMS module uses MamaNatalie for simulating prevention and management of PPH while the HBB module uses NeoNatalie for simulating neonatal resuscitation training and these trainings take one day for each module.

Standardising the BEmONC training is a commendable initiative, yet reaching to the great majority of providers with the training could be a daunting costly endeavour particularly for local stakeholders (such as health bureaus and health facilities) as they often have meagre resources. Hence, tailored in-service trainings to address conditions responsible for the deaths of most women and newborn babies would enhance responses. Short course in-service trainings such as the “one-day in-service HMS training for the management of PPH” and the “one day HBB training for neonatal resuscitation” which demonstrate substantial returns in reducing maternal and early neonatal mortality respectively [21, 28], could also be effective and efficient for Ethiopia to ensure providers competence in a short time period with limited budget.

The sample size would have been the main limitation of this report as the study assessed only 10 health facilities in Addis Ababa and included few providers. However, since the facilities were selected randomly and covered over one third of the HCs included in the 2008 EmONC survey, we believe that sample size and selection bias would be insignificant. Selecting the HCs randomly from each sub-city has improved representation, as all HCs under a sub-city would have similarity in infrastructure, logistics, in-service training and supervision. In this study, we used standard data collection tools and checklists adapted to the Ethiopian context to make a fair comparison with the 2008 national EmONC survey. Another limitation is that skills were assessed based on self-report not observed of what providers actually do during deliveries.

## Conclusions

Even though commendable progresses have been made in availing the necessary inputs and personnel, sub-optimal quality BEmONC services continues to prevail in Addis Ababa mainly due to poor providers’ competency, which should be a matter of urgent consideration. Although the standard national BEmONC curricula are comprehensive and up-to-date it appears to be not feasible for local partners such as health bureaus and health facilities to implement it. To further bridge the quality gaps in BEmONC, responsible stakeholders might need to consider tailoring competence based in-service short courses on priority maternal and neonatal conditions.

## Authors’ information

AHM is a postdoctoral fellow at the University of Bergen, Norway mainly working on health systems for maternal health, new born health and on the Prevention of mothers to child HIV transmission. MMS is working on behavioural health sciences at the School of Public Health, College of Health Sciences, Addis Ababa University, Ethiopia. ATR is MNCH team leader at JhPiego, Ethiopia. MMB is working for WAHA international and affiliated to the University of Gondar, Ethiopia.

## Abbreviations

AMTSL: Active Management of Third Stage Labour
BEmONC: Basic emergency obstetric and neonatal care
EmONC: Emergency obstetric and neonatal care
HC: Health center
FMOH: Federal Ministry of Health
MDG: Millennium development goal
PPH: Postpartum haemorrhage
WHO: World Health Organization.
